# Blood Cadmium Is Associated with Osteoporosis in Obese Males but Not in Non-Obese Males: The Korea National Health and Nutrition Examination Survey 2008–2011

**DOI:** 10.3390/ijerph121012144

**Published:** 2015-09-28

**Authors:** Won-Jun Choi, Sang-Hwan Han

**Affiliations:** Department of Occupational and Environmental Medicine, Gachon University Gil Medical Center, Incheon 21565, Korea; E-Mail: wjchoi@gachon.ac.kr

**Keywords:** cadmium, osteoporosis, obesity, men

## Abstract

Osteoporosis in males is becoming an important health concern in an aging society. The aim of this study was to investigate the associations between cadmium exposure and osteoporosis by considering the effect of obesity in aged males using a representative sample of the Korean population. Using the fourth and fifth Korea National Health and Nutrition Examination Survey data, 1089 males over 50 years of age were analyzed. The blood cadmium concentration was measured. The bone mineral density in the total hip, femur neck, and lumbar spine was measured using dual-energy X-ray absorptiometry. T-scores to determine the presence of osteoporosis were calculated using a Korean reference. Subjects were stratified into two groups according to obesity status (body mass index <25 kg/m^2^ and ≥25 kg/m^2^). In comparison with obese subjects with blood cadmium <1.00 μg/L, those with blood cadmium >1.50 μg/L had odds ratios of 4.57 (95% confidence interval [CI] 1.49–14.01) and 5.71 (95% CI 1.99–16.38) at the femur neck and any site, respectively, after adjusting for potential confounders such as age, serum creatinine, vitamin D deficiency, smoking, alcohol drinking, and physical activity level. However, this association was not significant in non-obese males. In conclusion, the effect of cadmium on osteoporosis was different by obesity status in aged males.

## 1. Introduction

Osteoporosis is a condition characterized by reduced bone mass and strength and increased risk of fracture [1]. Compared with the concern about bone health in postmenopausal females, osteoporosis in males has been overlooked. As the population ages, male osteoporosis and related issues, such as morbidity and mortality associated with osteoporotic fractures and medical costs, are becoming important public health problems [2].

Obesity causes or is associated with adverse health effects; however, there are epidemiologic studies that suggest a positive correlation between body weight or body mass index (BMI) and bone mineral density (BMD) [3,4]. Several explanations have been proposed. As the body mass increases, greater mechanical loads are imposed on bone. Consequently, the bone mass may increase to accommodate the greater load [5]. Increased estrogen production in obese postmenopausal women may be associated with the suppression of osteoclasts and increased BMD [6]. However, inconsistent findings also exist. Obesity is characterized by chronic inflammation with increased oxidative stress, and excessive oxidative stress may be one of the underlying mechanisms of osteoporosis [7,8].

Previous studies have been suggested that cadmium may be associated with adverse effects on bone health [9,10,11]. Findings from experimental research suggest the possible mechanistic pathways of the direct and indirect effects of cadmium on bone structure [12,13]. Although the exact mechanism is still uncertain, cadmium-induced oxidative stress may play an important role in the development of osteoporosis [14].

Although there have been several reports about the association between cadmium exposure and osteoporosis, few studies have focused on osteoporosis in older males and cadmium exposure. Furthermore, the effects of obesity and cadmium exposure have not been investigated simultaneously in previous studies. The aim of this study was to investigate the associations between cadmium exposure and osteoporosis by considering the effect of obesity in aged males using a representative sample of the Korean population.

## 2. Material and Methods

### 2.1. Data source and Study Subjects

This study was based on the fourth and fifth Korea National Health and Nutrition Examination Survey (KNHANES IV-V) data. The KNHANES is a regularly conducted cross-sectional study by the Korea Centers for Disease Control and Prevention. The KNHANES sample is representative of the Korean population. The KNHANES data are publicly available. The fourth KNHANES was conducted from 2007 to 2009, and the fifth KNHANES was conducted from 2010 to 2012. BMD and the blood cadmium level were measured from 2008, and the latest data disclosed to the public were for 2011. Thus, data from KNHANES 2008–2011 were used in this study. All of the KNHANES participants provided written informed consent before participation in the survey.

**Figure 1:**
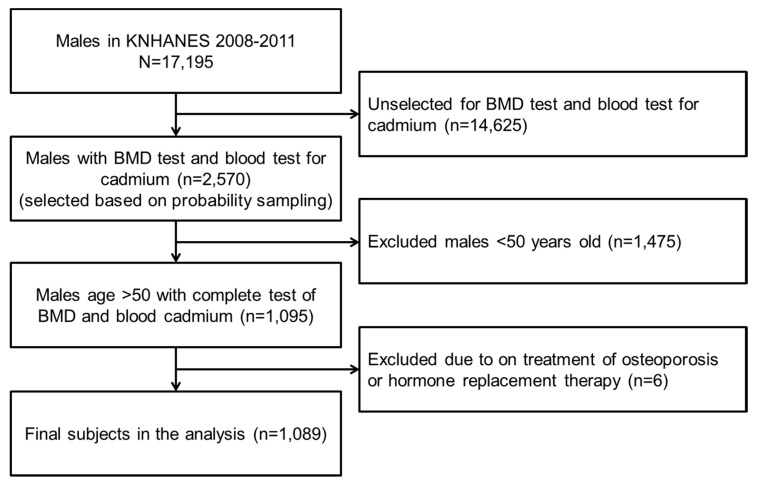
Flow diagram of subject inclusion and exclusion in the Korea National Health and Nutrition Examination Surveys (KNHANES) 2008–2011.

In total, 17,159 male subjects were identified. The BMD test and blood cadmium test were not performed for every subject, but only for 2000–2400 representative samples each year. In this dataset, there were 2570 male subjects who underwent a BMD test and blood test for cadmium simultaneously. Those aged under 50 years (*n* = 1475) were excluded. Individuals who were under treatment for osteoporosis or hormone replacement therapy were also excluded (*n* = 6). Ultimately, 1098 subjects were included in the analysis (Figure 1).

### 2.2. Measurement of BMD and Blood Cadmium

BMD was measured by dual energy X-ray absorptiometry (DXA) using the Discovery-W fan-beam densitometer (Hologic Inc., Bedford, MA, USA). BMD was measured at the total hip (left), femoral neck (left), and lumbar spine. When the BMD of the left femur could not be measured (e.g., due to surgery, fracture, deformity or malformation), the BMD of the right femur was measured. For the lumbar spine, the mean BMD of L1 to L4 was used. The T-score was used to determine osteoporosis in accordance with the recommendations of the WHO and ISCD. The T-score was calculated as follows:

$$\text{T}-\text{score}=\frac{\left(\text{individual BMD}-\text{refecence BMD}\right)}{\text{reference standard deviation of BMD}}$$

Reference BMD data were derived from a representative of healthy Korean adults aged 20–29 years. Osteoporosis was defined as a T-score less than −2.5 according to the WHO criteria.

Blood samples were collected from each subject to determine the blood cadmium and vitamin D concentrations. Blood samples for cadmium were collected using ethylene diamine tetra acetic acid (EDTA) tubes for trace elements. The blood samples were immediately processed, refrigerated, and transported in cold storage to the laboratory. The blood cadmium concentration was determined by graphite furnace atomic absorption spectrometry (GFAAS) using the PerkinElmer AAnalyst 600 (PerkinElmer, Turku, Finland) and presented as micrograms per liter. Traceability, the results of internal and external quality control were satisfied [15,16,17,18]. For example, 100 blood cadmium samples were double-checked in 2011 for testing traceability. External quality control assessment was performed six times per year (twice at German External Quality Assessment Scheme [G-EQUAS] and four times at CDC Lead and Multi-element Proficiency [CDC-Lamp]). Internal quality control assessment was performed four times per month using four test reagents with different concentrations, and most of all the results were within allowable range.

### 2.3. BMI and Other Potential Confounding Variables

BMI is presented as weight (in kilogram) divided by the square of height (in meters). Based on the West Pacific-Asian criteria of obesity, obesity was defined as BMI ≥25 kg/m^2^. The Vitamin D (25(OH)D) concentration was determined by a radioimmunoassay using a Gamma counter (Hewlett Packard, Meriden, CT, USA). Vitamin D deficiency was defined as 25(OH)D <20 ng/mL. Serum creatinine concentration, smoking (current smoker or non-smoker), excess alcohol drinking (>7 drinks of alcoholic beverages at a time, twice or more per week: yes or no), and physical activity (vigorous physical activity for more than 20 min at a time, three times or more per week: yes or no) were considered as potential confounders.

### 2.4. Statistical Analysis

Complex sampling design was adapted to the 4th and 5th KNHANES data. Participants were selected by stratified, clustered, and systematic sampling method to represent the population of Korea. In the statistical analysis, officially provided factors for strata, cluster, and weight were applied. The statistical analysis was performed using SAS 9.3 (SAS Institute, Cary, NC, USA). The SAS procedures for complex sample design such as proc surveymeans, proc surveyfreq, and proc surveylogistic were used with appropriate options.

Mean values (standard deviations [SD]) are presented for continuous variables, and percent (standard errors [SE]) is presented for categorical variables. The subjects were stratified into two categories by BMI. If the BMI was 25 kg/m^2^ or more, the subject was classified as obese. Logistic regression analysis was performed to determine the effect of cadmium on BMD. Blood cadmium concentrations were categorized into tertiles based on the distribution (<1.00 μg/L for the lowest tertile and >1.50 μg/L for the highest tertile). We built three models: an unadjusted model (model 1), a model adjusted for age (model 2), and a model fully adjusted for potential confounders (model 3). A *p*-value <0.05 was considered statistically significant.

## 3. Results

### 3.1. General Characteristics of the Subjects

In total, 1089 subjects were representatives for 1,909,528 males over 50 years of age (Table 1). The mean blood cadmium level was 1.25 μg/L (standard deviation, 0.66) and the geometric mean blood cadmium level was 1.09 μg/L (95% confidence limits, 1.04–1.14). The geometric means of blood cadmium according to tertile categories were as follows; 0.66 μg/L for lowest tertile (range 0.02–0.99), 1.22 μg/L for middle tertile (range 1.00–1.50), and 2.05 μg/L for highest tertile (range 1.51–6.04). The osteoporosis prevalence differed by body site: 3.8% at the total hip, 19.3% at the femur neck, 4.0% at the lumbar spine, and 20.7% at any of the above sites. The prevalence of obesity was 35.7%.

**Table 1:** General characteristics of the study subjects.

**Variables**		**n**		
No. of subjects (weighted frequency)		1089 (1,909,528)		
**Variables**		**Mean (SD ^(1)^)**	**Min**	**Max**
Age (years)		58.81 (7.47)	50	87
Blood cadmium (μg/L)		1.25 (0.66)	0.02	6.04
Serum creatinine (mg/dL)		0.96 (0.18)	0.60	2.70
25(OH)D (ng/mL)		21.22 (7.49)	4.85	47.01
**Variables**		**No. of Subjects**	**Weighted Frequency**	**% (SE ^(2)^)**
Osteoporosis (T-score < −2.5)	Total hip	46	72,353	3.8 (0.03)
	Femur neck	229	368,429	19.3 (0.07)
	Lumbar spine	46	76,254	4.0 (0.03)
	Any site	243	394,525	20.7 (0.07)
Obesity	BMI ≥25 kg/m^2^	368	682,304	35.7 (0.09)

**(1)** SD, standard deviation; **(2)** SE, standard error of mean.

### 3.2. BMD and T-Score by Obesity

The BMD and T-score of the total hip, femur neck, and lumbar spine by BMI are presented in Table 2. The T-score was calculated using the reference values for the Korean population. The BMD was higher and the T-score was lower in obese than in non-obese subjects at all body sites. The T-score of the femur neck was much lower than that of the total hip or lumbar spine.

**Table 2:** BMD and T-scores of the total hip, femur neck, and lumbar spine by obesity status.

BMI ^(1)^	Frequency	Weighted Frequency	Total Hip				Femur Neck				Lumbar Spine			
			BMD ^(2)^		T-Score ^(3)^		BMD		T-Score		BMD		T-Score	
			Mean	SD ^(4)^	Mean	SD	Mean	SD	Mean	SD	Mean	SD	Mean	SD
<25	721	1,227,224	0.91	0.12	−0.96	0.97	0.74	0.11	−1.76	1.06	0.92	0.14	−0.72	1.21
≥25	368	682,304	0.99	0.12	−0.29	0.97	0.80	0.11	−1.14	1.11	0.98	0.14	−0.17	1.24

**(1)** BMI, body mass index (kg/m^2^); **(2)** BMD, bone mineral density (g/cm^2^); **(3)** T-score, T-score using the Korean reference; **(4)** SD, standard deviation.

### 3.3. Association between the Blood Cadmium and Bone Mineral Density

The association between blood cadmium and bone mineral density was analyzed using regression models (Table 3). Blood cadmium was inversely associated with bone mineral density, but there was no significant association after adjusting for potential confounders in a saturated model (in model 3, regression coefficient −0.012 for total hip [*p* = 0.13]; −0.001 for femur neck [*p* = 0.88]; −0.001 for lumbar spine [*p* = 0.92]). There was a positive association between BMI itself and bone mineral density, but the effect size was relatively small (regression coefficient 0.017 for total hip [*p* < 0.01]; 0.013 for femur neck [*p* < 0.01]; 0.012 for lumbar spine [*p* < 0.01]).

**Table 3:** Effects of blood cadmium on bone mineral density **^(1)^** (results of regression analysis).

Body Site	Variables	Model 1 ^(4)^		Model 2 ^(5)^		Model 3 ^(6)^	
		Coefficient	*p*	Coefficient	*p*	Coefficient	*p*
Total hip	log Cd **^(2)^** (μg/L)	−**0.025**	**0.01**	**−0.021**	**0.01**	−0.012	0.13
	Age (years)			**−0.005**	**<0.01**	−**0.004**	**<0.01**
	BMI **^(3)^** (kg/m^2^)					**0.017**	**<0.01**
	Serum creatinine (mg/dL)					0.006	0.75
	Serum 25(OH)D (ng/mL)					0.001	0.06
	Current smoking					−0.004	0.66
	Alcohol drinking					0.006	0.55
	Physical activity					0.008	0.38
Femur neck	log Cd (μg/L)	−0.014	0.07	−0.009	0.21	−0.001	0.88
	Age (years)			−**0.005**	**<0.01**	−**0.005**	**<0.01**
	BMI (kg/m^2^)					**0.013**	**<0.01**
	Serum creatinine (mg/dL)					−0.022	0.26
	Serum 25(OH)D (ng/mL)					0.001	0.07
	Current smoking					−0.009	0.27
	Alcohol drinking					0.006	0.56
	Physical activity					0.003	0.74
Lumbar spine	log Cd (μg/L)	−0.011	0.23	−0.010	0.29	−0.001	0.92
	Age (years)			−**0.001**	**0.02**	−0.001	0.06
	BMI (kg/m^2^)					**0.012**	**<0.01**
	Serum creatinine (mg/dL)					**0.090**	**<0.01**
	Serum 25(OH)D (ng/mL)					0.001	0.82
	Current smoking					−0.012	0.28
	Alcohol drinking					0.002	0.89
	Physical activity					0.003	0.81

**(1)** Bone mineral density in g/cm^2^; **(2)** Cd, blood cadmium in log scale; **(3)** BMI, body mass index; **(4)** model 1, unadjusted model; **(5)** model 2, adjusted for age; **(6)** model 3, adjusted for age, BMI (as a continuous variable), serum creatinine, serum 25(OH)D, smoking (current smoker *vs.* non-smoker), alcohol drinking (>7 drinks of alcoholic beverage per time, twice or more in a week: yes or no), and physical activity (vigorous physical activity for more than 20 minutes per time, three times or more in a week: yes or no).

### 3.4. Association between the Blood Cadmium Level and Osteoporosis by Obesity Status

The association between blood cadmium and osteoporosis was analyzed using stratified logistic regression models (Table 4, detailed results by specific body site were presented in supplementary document). Compared with obese subjects with blood cadmium <1.00 μg/L, those with blood cadmium >1.50 μg/L had odds ratios (ORs) of 4.72 (95% confidence interval [CI], 1.98–11.28), and 4.81 (95% CI, 2.07–11.19) at the femur neck and any site, respectively (model 1, unadjusted model). With a saturated model (model 3), an increased risk of osteoporosis in obese males with a higher blood cadmium was still observed after adjusting for potential confounders (at the femur neck: OR, 4.57; 95% CI, 1.49–14.01; at any site: OR, 5.71; 95% CI, 1.99–16.38). A significant dose-response relationship was also observed (*p*-for-trend = 0.01). However, the association between blood cadmium level and osteoporosis was not significant in non-obese males.

The association between BMI (as a continuous variable) and osteoporosis was statistically significant in both groups, but the direction was opposite; an increment of BMI was associated with a decreased risk for osteoporosis in non-obese males (OR 0.71, 95% CI 0.64–0.79), but it was associated with an increased risk for osteoporosis in obese males (OR 1.26, 95% CI 1.01–1.58).

## 4. Discussion

In the present study, using a representative sample of the Korean population, we investigated the association between environmental cadmium exposure and osteoporosis in aged males, considering the obesity status. There was a significant association between the blood cadmium level and osteoporosis in obese males. A positive dose-response relationship was also observed. However, these associations were not significant in non-obese males.

Compared with women, particularly postmenopausal women, men are far less likely to be diagnosed with osteoporosis, and receive inadequate treatment for osteoporosis even after fracture [19,20]. Almost 30% of hip fractures occur in men [2], and mortality due to fracture appears to be higher in men than in women [21]. Although the exact pathogenesis of male osteoporosis is unclear, the role of estrogen in regulating bone density and bone turnover has been suggested in aged men [22,23]. Fracture risk also appears to be associated with sex hormones, such as estrogen and estradiol, in older men [24]. To reduce morbidity and mortality in older men, the importance of proper diagnosis and treatment for male osteoporosis should be emphasized.

Generally, the diagnosis of osteoporosis is based on the BMD measured by DXA, which is the most reliable predictor of fracture risk [25]. Osteoporosis is diagnosed if the T-score, which represents the number of standard deviations from the mean BMD in sex-matched young adults (*i.e.*, 20–29 years old), is −2.5 or less. Several factors such as gender, geographic factor and ethnicity may affect bone density. Although there have been reports regarding BMD differences among Asian countries [26,27], in Korea, the diagnosis of osteoporosis has been based on Asian references, based on Japanese individuals, not based on Korean young adults. Recently, new reference BMD values for the Korean population were reported [28]. The prevalence of osteoporosis in Korean males appears to be underestimated when Asian references are applied. In this study, we applied cutoff values using a population-based sample of Koreans to investigate the true effect of exposures on male bone density.

**Table 4:** Effects of blood cadmium on osteoporosis **^(1)^** (results of logistic regression analysis using the Korean reference for osteoporosis).

Strata	Variables	Prevalence of Osteoporosis			Model 1 ^(4)^			Model 2 ^(7)^			Model 3 ^(8)^		
		Frequency	Weighted Frequency	Prevalence (%)	OR ^(5)^	95% CI ^(6)^	*p*-for-Trend	OR	95% CI	*p*-for-Trend	OR	95% CI	*p*-for-Trend
BMI **^(2)^** <25 kg/m^2^	Cd (μg/L)^3)^												
	<1.00	78/281	126,359/487,263	25.9	Ref		0.98	Ref		0.93	Ref		0.22
	1.00~1.50	61/227	90,736/373,098	24.3	0.92	0.59 1.42		0.84	0.53 1.33		0.83	0.51 1.36	
	>1.50	59/213	96,126/366,862	26.2	1.01	0.64 1.60		0.99	0.61 1.61		0.72	0.42 1.23	
	Age (years)							**1.10**	**1.08 1.13**		**1.10**	**1.08 1.13**	
	BMI (kg/m^2^)										**0.71**	**0.64 0.79**	
	Serum creatinine (mg/dL)										1.48	0.54 4.06	
	Vitamin D deficiency										0.82	0.54 1.24	
	Current smoking										1.47	0.92 2.36	
	Alcohol drinking										1.13	0.62 2.08	
	Physical activity										1.11	0.65 1.90	
BMI ≥25 kg/m^2^	Cd (μg/L)												
	<1.00	15 /167	17,180/301,348	5.7	Ref		**<0.01**	Ref		**<0.01**	Ref		**0.01**
	1.00~1.50	12 /119	29,225/226,105	12.9	**2.46**	**1.01 6.01**		2.21	0.85 5.73		2.36	0.92 6.08	
	>1.50	18 / 82	34,900/154,850	22.5	**4.81**	**2.07 11.19**		**4.25**	**1.82 9.91**		**5.71**	**1.99 16.38**	
	Age (years)							**1.08**	**1.03 1.14**		**1.08**	**1.03 1.14**	
	BMI (kg/m^2^)										**1.26**	**1.01 1.58**	
	Serum creatinine (mg/dL)										0.56	0.07 4.35	
	Vitamin D deficiency										1.01	0.45 2.25	
	Current smoking										0.64	0.25 1.62	
	Alcohol drinking										0.58	0.20 1.70	
	Physical activity										0.65	0.26 1.64	

**(1)** Osteoporosis was defined as T-score < −2.5 at total hip, femur neck or lumbar spine; **(2)** BMI, body mass index (kg/m^2^); **(3)** Cd, blood cadmium concentration (lowest tertile (<1.00 μg/L) as a reference); **(4)** model 1, unadjusted model; **(5)** OR, odds ratio; **(6)** 95% CI, 95% confidence interval; **(7)** model 2, adjusted for age; **(8)** model 3, adjusted for age, BMI (as a continuous variable), serum creatinine (as a continuous variable), vitamin D deficiency (serum 25(OH)D <20 ng/mL), smoking (current smoker *vs.* non-smoker), alcohol drinking (>7 drinks of alcoholic beverage per time, twice or more in a week: yes or no) and physical activity (vigorous physical activity for more than 20 min per time, three times or more in a week: yes or no).

The adverse effect of cadmium on bone health has been reported [29,30]. The exact underlying mechanism of cadmium toxicity on bone has not been proved. Cadmium-induced bone damage may occur through both direct and indirect pathways. In the kidney, the inactive form of vitamin D (25(OH)D) is activated to the active form (1,25(OH)_2_D). Cadmium may interfere with the normal activation of vitamin D in the kidney. Calcium reabsorption may be decreased with renal tubular damage. Both vitamin D deficiency and hypercalciuria are associated with increased parathyroid hormone, which leads to bone loss by bone resorption to maintain the blood calcium level [31]. Furthermore, a direct effect of cadmium on the bone has also been suggested. *In vitro* and experimental animal studies have revealed that cadmium might decrease bone formation and increase bone resorption [32], and cadmium-induced bone demineralization appears to begin without renal damage [12]. Epidemiological studies have also reported that cadmium might decrease bone density without or independent of kidney damage [33,34].

There are two markers of cadmium exposure: urinary cadmium and blood cadmium. Urinary cadmium is believed to represent the body burden of cadmium, and blood cadmium is considered a marker of recent exposure. The biological half-life of cadmium is 10–30 years, which is mainly influenced by the concentration retained in the kidney. According to the previous report, the biological half-life of cadmium in the blood reflects two components: a rapid component of 3–4 months and a slow component of approximately 10 years [35]. Indeed, urinary cadmium and blood cadmium are significantly correlated with, rather than independent of, each other [29]. In addition, the blood cadmium level may also be a good estimate of the cadmium body burden, especially with long-term low-level exposure [36].

In the present study, the geometric mean concentration of blood cadmium was 1.09 μg/L, which appears to be lower than in residents of Beijing, China (geometric mean 1.23 μg/L for 46–60 years-old) [37] and higher than in Asians living in the US (geometric mean 0.39 μg/L) [38]. Several factors including age, dietary patterns, smoking status and other lifestyle factors might be influenced, and there is still a gap in knowledge and information regarding the differences in cadmium exposure level among different populations.

In this study, consistent with previous reports, an increased prevalence of osteoporosis was observed even at a low-level of cadmium exposure [9,29,30]. In this study, a significant association between the blood cadmium level and increased risk of osteoporosis was observed only at the femur neck. Although the reason is unclear, it is postulated that the femur neck may be one of the most vulnerable sites for the adverse effects of cadmium on BMD. Indeed, the BMD and T-score of the femur neck were lower than for the total hip and lumbar spine. Further research on the underlying mechanism is necessary.

In this study, we focused on the effect of cadmium on osteoporosis, considering the obesity status rather than the effect of obesity itself. There was a significant association between the blood cadmium level and osteoporosis in obese males only. Although the exact mechanism was not fully elucidated, a possible explanation might be that increased oxidative stress resulting from both obesity and cadmium exposure causes a lower bone density. Traditionally, obesity or a high body weight has been considered protective against osteoporosis [39,40]. However, this belief has recently been revised. Although increased mechanical loading may be beneficial to bone formation, obesity or excessive fat mass has been considered a risk factor for osteoporosis [41]. In this study, an increment of BMI was associated with increased risk of osteoporosis in obese males. It seems to be compatible with the results of recent studies. One of the possible mechanisms is that pro-inflammatory cytokines such as tumor necrosis factor and interleukin-6 may impair bone formation [42], suggesting that oxidative stress may adversely influence bone health [8,43]. Furthermore, cadmium-induced oxidative stress appears to be associated with bone damage [14]. With simultaneous exposure to cadmium and an obesity-induced inflammatory state, oxidative stress may be additively or synergistically increased. However, considering the cross-sectional design of this study, it cannot confirm or clearly explain the association between blood cadmium levels and the risk of osteoporosis in obesity. Experimental and longitudinal studies on the interaction effect of cadmium exposure and adiposity on bone health are required.

Using the KNHANES data, a representative sample of the Korean population, is one of the strengths of this study. Concern about potential bias seems to be minimal because the participants were selected using probability sampling method to represent the population. Moreover, we used the Korean reference for BMD to evaluate osteoporosis. With a consideration of ethnic differences, a more specific and precise interpretation would be possible.

There are several potential limitations in the present investigation. Firstly, because of the cross-sectional design, we cannot confirm the causal relationship. Secondly, BMI was used to define obesity in this study. It has been reported that the relationship between obesity and osteoporosis differs by how obesity is defined [44]. However, the aim of this study was to investigate the association between cadmium exposure and osteoporosis with respect to the obesity status, and we performed a stratified analysis by obesity status. There is no cutoff value of fat mass or a percentage of body fat for obesity. Thus, BMI appears to be a reliable index for the stratification of obesity status. Thirdly, potential underlying medical conditions might not be entirely excluded or controlled. However, a strict exclusion of subjects with comorbid conditions may cause the prevalence of osteoporosis to be underestimated [28]. We adjusted for serum creatinine in the saturated model, considering the relationship between kidney function and osteoporosis. Fourthly, a relatively small sample with low prevalence of outcome may lead to less confident inferences with wide confidence intervals. In this study, the data of four years (2008–2011) were aggregated to minimize this problem. However, the number of participants for blood cadmium test and bone mineral density test was relatively small because of survey feasibility. Few cases of osteoporosis at total hip and lumbar spine were related with wide confidence intervals. Even though the participants were selected based on the probability sampling to represent the population, lager sample might be necessary to infer more precisely.

## 5. Conclusions

In conclusion, the present study revealed a significant association between the blood cadmium level and the risk of osteoporosis in obese males. Defining vulnerable populations is an essential step to establish strategies for preventing public health problems. The results of this study suggest that environmental cadmium exposure might have more adverse effects on osteoporosis in susceptible populations, such as obese elderly individuals.

## Supplementary Files

Supplementary File 1
