# Supplementary material for: Blood Cadmium Is Associated with Osteoporosis in Obese Males but Not in Non-Obese Males: The Korea National Health and Nutrition Examination Survey 2008–2011
Source: Int J Environ Res Public Health. 2015 Sep 28;12(10):12144–57. doi: 10.3390/ijerph121012144 (PMC4626960; doi:10.3390/ijerph121012144)
Supplement: Supplementary File 1 [file ijerph-12-12144-s001.pdf]

Blood Cadmium Is Associated with Osteoporosis in Obese Males but not in Non-Obese Males: The Korea National Health and Nutrition Examination Survey 2008–2011

Table S1. Effects of blood cadmium on osteoporosis by body site (results of logistic regression analysis using the Korean reference for osteoporosis).

Body Site	Strata (BMI[1)])	Variables	Prevalence of Osteoporosis			Model 1 [(3)]			Model 2 [(6)]			Model 3 [(7)]		
			Frequency	Weighted Frequency	Prevalence (%)	OR [(4)]	95% CI [(5)]	*p*-for-Trend	OR	95% CI	*p*-for-Trend	OR	95% CI	*p*-for-Trend
Total hip	<25 kg/m²	Cd [(2)] (µg/L)												
		<1.00	14/281	20,124/487,263	4.1	Ref			Ref			Ref		
		1.00~1.50	11/227	16,661/373,098	4.5	1.09	0.44 2.67	0.32	1.00	0.40 2.51	0.34	0.85	0.29 2.47	0.90
		>1.50	15/213	23,119/366,862	6.3	1.57	0.67 3.69		1.59	0.64 3.97		1.08	0.32 3.70	
		Age (years)							**1.13**	**1.08 1.17**		**1.14**	**1.09 1.20**	
		BMI (kg/m²)										**0.60**	**0.50 0.72**	
		Serum creatinine (mg/dL)										3.44	0.89 13.37	
		Vitamin D deficiency										1.83	0.84 4.00	
		Current smoking										0.74	0.29 1.92	
		Alcohol drinking										1.36	0.38 4.94	
		Physical activity										1.38	0.49 3.91	
	≥25 kg/m²	Cd (µg/L)												
		<1.00	2/167	3,344/301,348	1.1	Ref			Ref			Ref		
		1.00~1.50	2/119	2,906/226,105	1.3	1.16	0.14 9.81	0.27	0.50	0.03 7.77	0.67	0.88	0.04 19.30	0.66
		>1.50	2/ 82	6,119/154,850	4.0	3.67	0.43 31.57		1.53	0.21 11.04		0.58	0.05 6.45	
		Age (years)							**1.25**	**1.15 1.36**		**1.27**	**1.16 1.39**	
		BMI (kg/m²)										0.54	0.29 1.01	
		Serum creatinine (mg/dL)										0.18	0.01 47.32	
		Vitamin D deficiency										1.61	0.21 12.50	

Table S1. *Cont.*

Body Site	Strata (BMI [1])	Variables	Prevalence of Osteoporosis			Model 1 [3]			Model 2 [6]			Model 3 [7]		
			Frequency	Weighted Frequency	Prevalence (%)	OR [4]	95% CI [5]	*p*-for-Trend	OR	95% CI	*p*-for-Trend	OR	95% CI	*p*-for-Trend
		Current smoking										4.08	0.22 74.60	
		Alcohol drinking										-	-	
		Physical activity										-	-	
Femur neck	<25 kg/m^2	Cd (µg/L)												
		<1.00	75/281	122,646/487,263	25.2	Ref			Ref			Ref		
		1.00~1.50	57/227	82,968/373,098	22.2	0.85	0.55 1.33	0.94	0.76	0.48 1.22	0.84	0.75	0.45 1.24	0.18
		>1.50	56/213	91,930/366,862	25.1	0.99	0.62 1.59		0.97	0.59 1.60		0.69	0.39 1.20	
		Age (years)							**1.11**	**1.08 1.13**		**1.11**	**1.08 1.14**	
		BMI (kg/m^2)										**0.70**	**0.63 0.78**	
		Serum creatinine (mg/dL)										1.70	0.61 4.72	
		Vitamin D deficiency										0.78	0.51 1.20	
		Current smoking										1.43	0.89 2.30	
		Alcohol drinking										1.21	0.65 2.28	
		Physical activity										1.05	0.60 1.83	
	≥25 kg/m^2	Cd (µg/L)												
		<1.00	14/167	16,414/301,348	1.1	Ref			Ref			Ref		
		1.00~1.50	10/119	21,374/226,105	1.3	1.81	0.70 4.72	**<0.01**	1.55	0.56 4.30	**<0.01**	1.78	0.62 5.14	**0.01**
		>1.50	17/ 82	33,098/154,850	4.0	**4.72**	**1.98 11.28**		**4.10**	**1.69 9.98**		**4.57**	**1.49 14.01**	
		Age (years)							**1.10**	**1.04 1.17**		**1.10**	**1.04 1.16**	
		BMI (kg/m^2)										0.96	0.72 1.28	
		Serum creatinine (mg/dL)										0.72	0.08 6.14	
		Vitamin D deficiency										0.81	0.35 1.89	
		Current smoking										0.78	0.29 2.10	
		Alcohol drinking										0.38	0.10 1.48	

Table S1. *Cont.*

Body Site	Strata (BMI [1])	Variables	Prevalence of Osteoporosis			Model 1 [3]			Model 2 [6]			Model 3 [7]		
			Frequency	Weighted Frequency	Prevalence (%)	OR [4]	95% CI [5]	*p*-for-Trend	OR	95% CI	*p*-for-Trend	OR	95% CI	*p*-for-Trend
		Physical activity										0.44	0.15 1.27	
Lumbar	<25 kg/m^2	Cd (μg/L)												
Spine		<1.00	12/281	18,923/487,263	3.9	Ref			Ref			Ref		
		1.00~1.50	15/227	24,243/373,098	6.5	1.72	0.71 4.18	0.41	1.66	0.69 4.04	0.43	1.44	0.59 3.51	0.96
		>1.50	12/213	20,163/366,862	5.5	1.44	0.55 3.75		1.43	0.55 3.73		1.03	0.41 2.60	
		Age (years)							**1.05**	**1.01 1.10**		**1.06**	**1.01 1.11**	
		BMI (kg/m^2)										**0.80**	**0.67 0.95**	
		Serum creatinine (mg/dL)										0.47	0.09 2.48	
		Vitamin D deficiency										1.62	0.78 3.37	
		Current smoking										1.72	0.69 4.30	
		Alcohol drinking										1.22	0.39 3.86	
		Physical activity										1.62	0.62 4.24	
	≥25 kg/m^2	Cd (μg/L)												

Table S1. *Cont.*

Body Site	Strata (BMI (1))	Variables	Prevalence of Osteoporosis			Model 1 (3)			Model 2 (6)			Model 3 (7)		
			Frequency	Weighted Frequency	Prevalence (%)	OR (4)	95% CI (5)	*p*-for-Trend	OR	95% CI	*p*-for-Trend	OR	95% CI	*p*-for-Trend
		<1.00	2/167	1,578/301,348	0.5	Ref			Ref			Ref		
		1.00~1.50	4/119	9,544/226,105	4.2	**8.37**	**1.27 55.19**	0.16	**8.61**	**1.25 59.43**	0.13	4.41	0.79 24.56	0.44
		>1.50	1/ 82	1,802/154,850	1.2	2.24	0.20 25.14		2.37	0.21 26.26		2.18	0.12 40.11	
		Age (years)							0.97	0.85 1.11		0.99	0.88 1.11	
		BMI (kg/m^2)										**1.69**	**1.33 2.16**	
		Serum creatinine (mg/dL)										1.26	0.13 12.57	
		Vitamin D deficiency										2.79	0.45 17.40	
		Current smoking										0.68	0.19 2.41	
		Alcohol drinking										1.10	0.41 2.94	
		Physical activity										1.21	0.30 4.86	

(1) BMI, body mass index (kg/m^2); **(2)** Cd, blood cadmium concentration (lowest tertile (<1.00 µg/L) as a reference); **(3)** Model 1, unadjusted model; **(4)** OR, odds ratio; **(5)** 95% CI, 95% confidence interval; **(6)** Model 2, adjusted for age; **(7)** Model 3, adjusted for age, BMI (as a continuous variable), serum creatinine (as a continuous variable), vitamin D deficiency (serum 25(OH)D <20 ng/mL), smoking (current smoker vs non-smoker), alcohol drinking (>7 drinks of alcoholic beverage per time, twice or more in a week: yes or no) and physical activity (vigorous physical activity for more than 20 min per time, 3 times or more in a week: yes or no).

© 2015 by the authors; licensee MDPI, Basel, Switzerland. This article is an open access article distributed under the terms and conditions of the Creative Commons Attribution license (http://creativecommons.org/licenses/by/4.0/).
